# A transversal cross-sectional study of factors related to HPV vaccination status and cancer screening participation among French women aged 25–40

**DOI:** 10.1186/s12885-024-12591-1

**Published:** 2024-07-06

**Authors:** Fanny Serman, Gabrielle Lisembard, Maxence Sahraoui, Christophe Berkhout, Michaël Rochoy, Anthony Haro, Matthieu Calafiore

**Affiliations:** 1 Department of General Medicine, School of Medicine, Lille University, Lille, France; 2 ULR 2694 – METRICS, Lille University, Lille, France

**Keywords:** Uterine cervical cancer, Early detection of cancer, HPV (Human Papilloma Virus), Epidemiologic study

## Abstract

**Background:**

In 2020, uterine cervical cancer (UCC) was the 12th most common cancer among women in France and the 4th worldwide. French health authorities wanted to increase Human *Papilloma* Virus (HPV) vaccination and screening rates. There were still many barriers to these measures among young women, their families, and health professionals and teachers. Between 2014 and 2019, international studies found inconsistent effects of HPV vaccination on UCC screening. In 2022, a survey was conducted among women aged 25 to 40 in the Nord-Pas-de-Calais region to assess participation 1) in HPV vaccination and its barriers, 2) in UCC screening as a function of HPV vaccination status.

**Methods:**

Data were collected using an anonymous online questionnaire distributed by QR code in 80 general practices randomly selected in the Nord-Pas-de-Calais region between January and June 2022. Results were analyzed bivariately using the Chi2 test, multivariately when numbers allowed, and in age subgroups (sensitivity analysis).

**Results:**

407 complete questionnaires (for 602 participating women) were analyzed. In our sample, 41% of women aged 25 to 40 in the Nord-Pas-de-Calais region were vaccinated against HPV viruses in 2022. The risk factors for non-vaccination, after multivariable adjustment, were: the periods of eligibility for vaccination in the early days of French vaccination (2007–2012: odds ratio OR = 0.04 [95% CI, 0.02–0.09]; 2012–2017: OR = 0.5 [0.3–0.8]), information received from non-medical sources (OR = 0.3 [0.2–0.6]), and absence of information about vaccination (OR = 0.12 [0.05–0.27]). In our sample, 90% of women were screened for UCC. In bivariate analysis, women at risk of not being screened were those who were youngest, had been vaccinated against HPV, were not heterosexual, lived alone, had gynecological follow-up by their general practitioner, and did not have regular gynecological follow-up. Sensitivity analysis showed that the only risk factor significantly correlated with non-screening regardless of age group was lack of regular gynecological follow-up.

**Conclusions:**

Participation in HPV vaccination and UCC screening is improved by medical education and gynecological follow-up. This multicenter study, limited by the relative youth of vaccination in France, should be repeated after 2037 to assess the possible effect of vaccination on screening.

## Background

In 2020, uterine cervical cancer (UCC) will be the twelfth most common cancer among women in France and the fourth most common cancer worldwide [1], making it a major public health concern. Incidence and mortality rates for UCC have declined steadily in France since the introduction of cervical screening by cervical cytology (Pap smear) in the 1960s [2]. However, this decline has slowed, probably due to increased exposure to high-risk human papillomaviruses (HPV-HR) associated with changes in sexual behavior, such as a decrease in the age of sexual debut and an increase in the number of sexual partners [3, 4]. By 2020, 90% of new cases and deaths will occur in developing countries [1]. According to the World Health Organization (WHO), by 2022, UCC will be considered a preventable and eliminable cancer through preventive measures. These measures, vaccination, and cervical cytology screening, were more accessible in developed countries. The World Health Assembly adopted a strategy to eliminate UCC as a public health problem and set the "90–70-90" targets. By 2030, this would mean that 90% of girls should be fully vaccinated by age 15, 70% of women should be screened at ages 35 and 45, and 90% of women diagnosed with cervical disease (precancerous lesions or invasive cancer) should receive treatment [5].

In France, the history of HPV vaccination is complex. It started in 2007 targeting 14-year-old girls, with catch-up vaccination for women aged 15 to 25. In 2012, it was extended to girls aged 11 to 14, with catch-up for those aged 15 to 19. In 2019, vaccination was recommended for men under 26 who have sex with men. Since 2021, it is recommended for all genders aged 11 to 19. Cervical cancer screening in France was done by cytological examination of a cervical smear every three years for women aged 25 to 65 until 2019. The widespread adoption of HPV molecular detection changed this in 2019 for women aged 30 to 65, shifting to HPV testing on cervical smears every five years (remaining unchanged for those aged 25 to 30). Despite the availability of these two complementary preventive measures, vaccination coverage and screening rates remained insufficient. Acceptance and adherence to HPV vaccination have been described as a complex concept involving users, parents, and professionals, which is improved by talking about the vaccine and about sexuality [6]. In Italy in 2022, HPV vaccination coverage was clearly inadequate, as was adherence to screening, both far from WHO targets [7]. Studies have identified perceived barriers to vaccination among the student population[8] as well as among health care workers and teachers[9], including mistrust of the vaccine due to its perceived novelty, low knowledge, and misinformation about the vaccine. A Chinese study found in 2015 that educational intervention on HPV increased awareness regarding HPV and women’s intention to vaccinate themselves[10]. Several studies, including one from Belgium and one from France, have shown that young girls who are vaccinated are more likely to have a mother who is regularly screened. Preventive behaviors tend to cluster within families [11, 12]. Our first objective was to describe the HPV vaccination prevalence in the Nord-Pas-de-Calais departments in France and to assess potential risk factors for non-vaccination.

The second concern that could arise is participation in UCC screening, especially among vaccinated women, since even general practitioners in France in 2020 feared that vaccination would reduce participation in screening [13]. In Denmark and the United Kingdom, vaccinated women would be more likely to undergo screening [14, 15]. In a French study conducted in 2011 among women aged 25–65, 66.7% of respondents believed that vaccinated women should continue to undergo cervical screening by Pap smear, while 29.1% said they did not know. However, this study did not consider vaccination status [16]. Internationally, several studies have attempted to examine the association between HPV vaccination status and screening uptake. Most of these studies were conducted in Australia or Japan, where organized screening programs have been in place for several years, and in the United States. They showed statistically significant higher screening rates among vaccinated women compared with unvaccinated women [17–19]. European studies have also found similar results, including in Sweden and Italy [20, 21]. Only an Australian study from 2014 showed a statistically significant lower screening rate among vaccinated women [22]. To our knowledge, there have been no other studies in France, and particularly in Nord-Pas-de-Calais, where cervical cancer screening rates were barely 60%, on screening participation based on vaccination status in 2022. The primary objective was to determine if there was a significant association between HPV vaccination status and cervical cancer screening among eligible women in Nord-Pas-de-Calais.

The secondary objectives were to determine the sociodemographic and medical characteristics, the modalities of use of vaccination and screening, and the knowledge of these two means of prevention among the responding women according to their HPV vaccination status.

## Methods

### Design

The epidemiologic study was a retrospective analytical observational study conducted between February 1 and June 1, 2022. It was a multicenter study conducted in the Nord-Pas-de-Calais region (France) using an online self-administered questionnaire targeting women aged 25–40 years. The questionnaire was made accessible through a QR code placed in the waiting rooms of randomly selected general practitioners' offices.

### Population

The study population consisted of French women aged 25–40 years who visited randomly selected general practitioners in Nord and Pas-de-Calais and completed the questionnaire. Exclusion criteria were: women under 25 or over 40 years of age, incomplete questionnaires, women who did not know their HPV vaccination status, women who did not know their cervical cancer screening status. We excluded women above 40 in 2022 as they had not had access to the vaccination (the French vaccination program began in 2007 for young women aged 9–25 years), and women under 25 since they were not targeted by the screening program. The minimum number of analyzable questionnaires required to achieve adequate power was 385.

GP’s were chosen for their centrality in the primary care network to ensure a broad and representative sample that was homogeneously distributed and practically feasible without additional resources. The authors' experience was that about 15% of their colleagues agreed to participate in clinical studies by displaying a survey request in their waiting room, resulting in 5 to 10 responses over a period of 3 months: a random sample of 400 GPs was selected from the exhaustive list of 3,780 general practitioners (GPs) in Nord and Pas-de-Calais. These GPs were contacted by telephone in January 2022 and 80 agreed to distribute the questionnaire. They displayed an A4 poster with a brief explanatory text and the QR code to access the online questionnaire (via Limesurvey), as well as A7 cards containing the QR code.

### Questionnaire

The questionnaire was created in the most comprehensive way possible from a narrative literature review on risk factors for non-HPV vaccination and non-screening for cervical cancer. It was tested twice by ten women aged 25–40 years (not included in the study), with rewriting of any unclear or ambiguous questions, and logical organization into subsections. It was divided into the following parts: Part 1: Sociodemographic characteristics of the women; Part 2: Behaviors, history, and information received regarding HPV and non-HPV vaccinations; Part 3: Behaviors, history, and information received regarding gynecologic follow-up and cervical cancer screening with the Papanicolaou test; Part 4: About their primary health care provider; Part 5: Women's knowledge of vaccination and screening.

### Definition of Variables

The variables 'vaccination' and 'participation in screening' were binary. Any woman who reported having received at least one dose of vaccine was considered vaccinated. Any woman who reported having had a Pap smear within the past three years or in the past three years was considered screened. Any woman who reported having had a Pap smear more than three years ago or who reported never having had a Pap smear was considered unscreened.

The variable "knowledge about cervical cancer screening" was a score (from 0 to 4) constructed as the sum of correct answers to 4 questions about screening: 1) In your opinion, is a woman who has been vaccinated against HPV protected against all types of human papillomavirus? [good answer: "no"] 2) In your opinion, is screening necessary if a woman is vaccinated against HPV? [good answer: "yes"] 3) In your opinion, is it recommended to be screened for cervical cancer with a Pap smear? [good answer among 5: "from the age of 25 to 65"] 4) In your opinion, the purpose of a Pap smear is? [2 answers required out of 5: "to detect abnormal cells in the pre-cancerous stage", "to detect the presence of human papillomavirus"].

### Qualitative variables analysis

Reasons for non-participation in vaccination or screening were provided by participants in free text. Semantic analysis allowed for qualitative exploration of participants' responses.

### Statistical methods

The population of women aged 25 to 40 in Nord-Pas-de-Calais was approximately 375,000 on January 18, 2022. The minimum number of analyzable questionnaires required to achieve adequate power was 385, with a selected alpha risk of 5%. Standard errors for descriptive statistics of the entire population were calculated at the 95% confidence level [23]

Categorical variables were expressed as frequencies and percentages. Independence (or correlation) between two qualitative variables was tested using chi-squared tests. Fisher's exact test was used when theoretical frequencies were less than 5. Descriptive statistics were performed with Excel®. Bivariate statistical analyses were performed using jamovi 2.4.11.0 [24, 25] with a selected alpha risk of 5%.

Multivariable analyses for HPV vaccination status were performed using logistic regression, with the outcome variable "HPV vaccination status" and the explanatory variables "HPV information provider" and "area of residence". Candidate covariates were included in a penalized Least Absolute Shrinkage and Selection Operation (LASSO) model in order to provide a more robust logistic regression. The penalty coefficient (lambda) was chosen to provide an estimation error less than one standard deviation of the minimum error obtained by tenfold cross-validation, while being as parsimonious as possible. No variable had a coefficient different from 0 with this lambda coefficient [26].

### Ethics

This study was the subject of a declaration to the Data Protection Officer of the University of Lille with an agreement received on January 12, 2022 (n° 2022–013) corresponding to the start of the study. The participation of physicians and women was voluntary. All data were collected anonymously and in accordance with the recommendations of the data protection officer. This work received no public or private funding.

## Results

### Sociodemographic characteristics

- Study centers: 80 general practitioners (out of 400 randomly selected) agreed to be study centers. Of these, 62.5% were in the Nord department and 37.5% in the Pas-de-Calais department. Of these, 62.5% were men and 17.5% were university trainers of general practice residents. Study centers were homogeneously distributed on the territory.
-Study population: Of the 80,000 patients in general practices in Nord-Pas-de-Calais, 602 women responded. Excluded were 98 incomplete questionnaires, 64 participants under 25 or over 40, 32 questionnaires (5,3%) where HPV vaccination status was unknown, 1 questionnaire (0,2%) where cervical cancer screening status was unknown.

Table 1 presents the sociodemographic descriptive statistics of the 407 participants enrolled.

**Table 1:** Respondent characteristics

Characteristics of respondents	n	%
**Age ranges**		
25–29 years	144	35,4
30–34 years	121	29,7
35–40 years	142	34,9
**Socioprofessional category**		
Farmers	0	0,0
Craftsmen, shopkeepers, company managers	15	3,7
Executives and higher intellectual professions	144	35,4
Intermediate professions	44	10,8
Employees	146	35,9
Manual workers	10	2,4
Inactive who have already worked	35	8,6
Never worked	13	3,2
**Location**		
In town	289	71,0
In the country	118	29,0
**Department**		
Nord	285	70,0
Pas-de-Calais	122	30,0
**Marital status**		
Single	58	14,3
Cohabiting	103	25,3
Civil union	91	22,4
Married	141	34,6
Separated	6	1,5
Divorced	7	1,7
Widowed	1	0,2
**Sexual relations**		
Hetero	377	92,6
Homo	11	2,7
Bi	11	2,7
No sexual relations	8	2,0
**Referring physician**		
Men	225	44.7
Female	182	55.3
Performing pap smears	104	25.6
Not performing pap smears	150	36.9
Unknown	153	37.6
Working alone	159	39.1
Practicing in a group	233	57.2
Not known	15	3.7

### Comparison of the characteristics of vaccinated and unvaccinated women

40.8% of respondents were vaccinated against HPV viruses. Table 2 compares the characteristics (theoretically prior to their HPV vaccination) of vaccinated and unvaccinated participants.

**Table 2:** Comparison of respondent characteristics by HPV vaccination status. HBV Hepatitis B virus. Fisher test when theoretical numbers < 5; Chi2 test

Characteristics	Vaccinated n (%)	Unvaccinated n (%)	*p* value
**Total** ***n*= 407**			
	166 (40,8)	241 (59,2)	
**Age ranges**			
25–29 years	98 (68,1)	46 (31,9)	< 0,001
30–34 years	60 (49,6)	61 (50,4)	
35–40 years	8 (5,6)	134 (94,4)	
**Location**			
City	128 (44,3)	161 (55,7)	0,02
Countryside	38 (32,2)	80 (67,8)	
**Department**			
Nord	119 (41,8)	166 (58,2)	0,54
Pas-de-Calais	47 (38,5)	75 (61,5)	
**HBV vaccination status**			
Vaccinated	120 (40,1)	179 (59,9)	0,15
Unvaccinated	13 (28,9)	32 (71,1)	
**Information on HPV vaccination by**			
Healthcare professional	138 (58,7)	97 (41,3)	< 0,001
Entourage/Media/Internet	21 (21,6)	76 (78,4)	
No information	7 (9,3)	68 (90,7)	

Vaccinated and unvaccinated women had similar characteristics except for age (i.e., year of vaccination), place of residence, and source of information about HPV vaccination. Women in the 35–40 age group (targeted for HPV vaccination in 2007–2010) were the least vaccinated (5.6%). Women in the 30–34 age group targeted for vaccination in 2007–2011 were less vaccinated (49.6%) than women in the 25–29 age group targeted for vaccination in 2007–2016 (68.1%). This difference was statistically significant (*p* < 0.001). Women living in cities were more likely to be vaccinated (44.3%) than women living in rural areas (32.2%), and this difference was also statistically significant (*p* = 0.02). Women who had received information about HPV vaccination from a health professional (124 from a general practitioner, 14 from a gynecologist) were significantly more likely to have been vaccinated.

Women vaccinated against HBV were more likely to be vaccinated against HPV, but this difference was not statistically significant (*p* = 0.15).

**Fig. 1:**
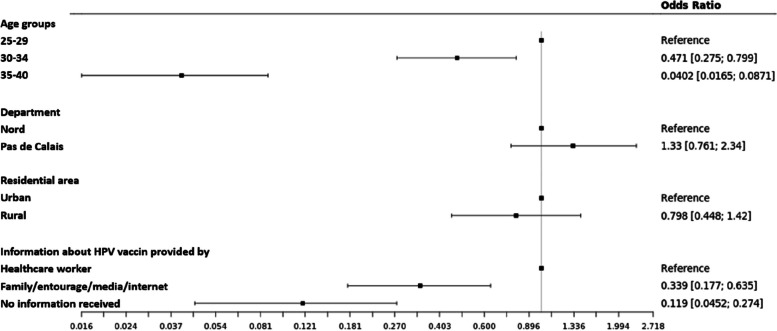
Multivariate analysis of risk factors for HPV non-vaccination

In multivariate analysis, women who had received information about HPV vaccination from a healthcare provider were significantly more likely to be vaccinated, after adjustment for age group and residence. Figure 1 shows the odds ratio of these three variables in relation to HPV vaccination status.

### Reasons given for non-vaccination

The reasons given in the free fields ("not wanted" and "other") could be grouped into several categories: “I am over the age” 18 women; “no hindsight/doubt/start of vaccination” 15 women; “parents are against it” 14 women; “don't know what it is for/methods/not enough information” 8 women; “doesn't feel concerned” 7 women; “too late for sexual debut” 5 women; “vaccine not mandatory” 2 females.

### Comparison of the characteristics of women taking part in UCC screening

Table 3 shows all the factors that characterize screened women compared to unscreened women.

**Table 3:** Characteristics of women screened

Characteristics	Women screened n (%)	*p* value
**Total** ***n*=407**	364 (89,4)	
**HPV vaccination status**		
Vaccinated	141 (84,9)	0,01
Unvaccinated	223 (92,5)	
**Age ranges**		
25–29 years	117 (81,2)	< 0,001
30–34 years	113 (93,4)	
35–40 years	134 (94,4)	
**Socioprofessional categories**		
Farmers	0	0,98
Craftsmen, shopkeepers,	14 (93,3)	
Executives and higher intellectual professions	128 (88,9)	
Intermediate professions	40 (90,9)	
Employees	131 (89,7)	
Manual workers	9 (90,0)	
Inactive having already worked	30 (85,7)	
Never worked	12 (92,3)	
**Location**		
City	255 (88,2)	0,22
Countryside	109 (92,4)	
**Department**		
Nord	255 (89,8)	0,97
Pas-de-Calais	109 (89,3)	
**Marital status**		
Living together	92 (89,3)	< 0,01
Civil union partner	84 (92,3)	
Married	130 (92,2)	
Single	47 (81,0)	
Separated	4 (66,7)	
Divorced	7 (100,0)	
Widowed	0	
**Sexual orientation**		
Hetero	344 (91,2)	< 0,001
Homo	7 (63,6)	
Bi	9 (81,8)	
No sexual relations	4 (50,0)	
**Frequency of gynecological check-ups**		
Every 6 months	24 (96,0)	< 0,001
Every year	237 (95,6)	
Every 2 years	75 (92,6)	
Every 3 years or more	25 (67,6)	
No follow-up	3 (18.8)	
**Professional who performs gynecological check-ups**		
MG	18 (75,0)	0,003
SF	87 (91,6)	
Gynecologist	256 (94,1)	
**Information on cervical cancer screening by**		
Healthcare professional	240 (91,6)	0,17
Surroundings/Media/Internet	83 (87,4)	
No information	41 (83,7)	
**Attending physician**		
Men	196 (87,1)	0,09
Female	168 (92,3)	
Performing pap smears	90 (86,5)	0.53
Not performing pap smears	136 (90,7)	
Working alone	143 (89,9)	0.48
Practicing in a group	209 (89,7)	
**Knowledge associated with screening (score)**		
0 No knowledge	20 (87.0)	0.21
1	67 (89.3)	
2	149 (93.7)	
3	97 (85.1)	
4 Excellent knowledge	31 (86.1)	

In bivariate analysis, screening rates were lower among women vaccinated against HPV viruses, younger women, single or separated women, and homosexual or asexual women. Vaccinated women screened less (15.1% nonparticipation) than unvaccinated women (7.5% nonparticipation), and this was statistically significant (*p* = *0.01*). Women in the 30–34 and 35–40 age groups were significantly more likely to be screened (93.4% and 94.4%, respectively) than women in the 25–29 age group (81.2%) (*p* < *0.001*). There was no significant difference according to socio-professional category (*p* = *0.99*), department (*p* = *0.97*), or place of residence (*p* = *0.22*).

As the frequency of gynecologic follow-up decreased, the screening rate also decreased significantly (*p* < *0.001*). The screening rate is significantly higher when the follow-up is performed by a midwife (91.6%) or a gynecologist (94.1%) than by a general practitioner (75%) (*p* = *0.003*).

The low rate of unscreened women in our sample precluded multivariate analysis of the effect of vaccination status on screening rates. A sensitivity analysis was performed to assess whether the association remained significant in age subgroups (shown in Table 4).

**Table 4:** Risk factors for non-screening of UCC by age subgroup. In each subgroup n provides the absolute number of screened women in one category and % provides the percentage of screened women in this category (for example 78 vaccinated women aged 25–29 were screened and they represent 79,6% of all 98 vaccinated 25–29 women)

	**25–29 age group** **(*n*= 144)** **Women screened**		**30–34 age group** **(*n*=121)** **Women screened**		**35–40 age group** **(*n*=142)** **Women screened**	
**Total n(%)**	117 (81,2)		113 (93,4)		134 (94,4)	
**Characteristics**	n (%)	*p* value	n (%)	*p* value	n (%)	*p* value
**Vaccination status**						
Vaccinated	78 (79,6)	0,46	55 (91.7)	0.45	8 (100)	0.48
Unvaccinated	39 (84,8)		58 (95.1)		126 (94.0)	
**Marital status**						
In couple	91 (84,3)	0,11	97 (94,2)	0.41	118 (95,2)	0.28
Not a couple	26 (72,2)		16 (88,9)		16 (88,9)	
**Sexual orientation**						
Heterosexual	112 (85,5)	** < 0,001**	106 (93,8)	0.49	126 (94,7)	0.47
Bi/homo/a-sexual	5 (38,5)		7 (87,5)		8 (88,9)	
**Frequency of gynecological check-ups**						
Every 6 months	12 (92,3)	** < 0,001**	4 (100)	** < 0,001**	8 (100)	**< 0,001**
Yearly	70 (90,9)		71 (95,9)		96 (99)	
Every 2 years	24 (82,8)		28 (100)		23 (95,8)	
Every 3 years or more	9 (69,2)		9 (75)		7 (58,3)	
No follow-up	2 (16,7)		1 (33,3)		0 (0)	
**Professional who performs gynecological check-ups**						
MG	8 (80)	0,58	8 (80)	0.08	2 (50)	**< 0,001**
SF	32 (84,2)		31 (96,9)		24 (96)	
Gynecologist	75 (89,3)		73 (96,1)		108 (96,4)	

Sensitivity analysis showed that after adjusting for age, being screened was not significantly correlated with being vaccinated against HPV viruses. The only risk factor significantly correlated with non-screening regardless of age group was lack of regular gynecological follow-up.

### Reasons given for not screening

20 women (4.9%) had never received pap smears. Of these, 95% were between the ages of 25 and 29. 23 women (5.6%) had received pap smears but were no longer up to date. Among the women who had never had a pap smear, the most common reasons for not having it were: “not wanted” (5 women, 3 of whom had been vaccinated), “not yet done because they were in their 25th year” (4 women, 3 of whom had been vaccinated), “problems with access to gynecological care” (3 women, 2 of whom had been vaccinated), “forgotten” (2 women, 1 of whom had been vaccinated), “not suggested by a health professional” (2 women, 1 of whom had been vaccinated).

## Discussion

### Vaccination against HPV

In 2022, 41 ± 5% of French women aged 25–40 years in the Nord-Pas-de-Calais region were vaccinated (at least one dose) against HPV viruses. This corresponds to the official epidemiologic figures for this age group in France [27]. The risk factors for non-vaccination, after adjustment, were the period of eligibility for vaccination in the early days of vaccination in France, information received from non-medical sources, or lack of information about vaccination. Qualitative reasons given by women who had not been vaccinated were mainly that they were older than the age for vaccination, that they doubted the safety of the vaccine, and that their parents were against vaccination.

In bivariate analysis, the HPV vaccination rate was lower in rural areas (32.2%) than in urban areas (44.3%), but this difference was no longer significant after adjustment. Several studies (international [28–30] and French [31, 32]) found that more women were vaccinated in urban areas. This could be explained by accessibility in terms of distance and frequency of medical centers, especially as a shorter distance between home and vaccination site was significantly associated with a better HPV vaccination rate in the Netherlands [33]. However, there appeared to be no difference in vaccine acceptance between rural and urban areas [34]. There was also no difference in vaccine recommendation between general practitioners practicing in rural and urban areas [35].

The main source of information about vaccination was medical (57 ± 5%), with GPs accounting for 80%. GPs were identified as the main source of information in 46% of cases, all sources combined. These results are similar in the general population, as shown by a survey conducted by INCa and HAS in 2019, in which 86% of parents identified their GP as their main source of information on this topic. Moreover, in the same study, 96% of GPs stated that they were in favor of vaccination, but only 40% systematically offered it, fearing a refusal that could lead to conflict [27]. Similarly, in our study, only 46% of women (themselves or their parents) were offered vaccination by their GP. The provision of information by the general practitioner seems essential to increase the knowledge of parents and young girls, to improve their understanding of the issues, and thus to increase their acceptance of the vaccination. This is confirmed by the first reason for non-vaccination, “not suggested by a health professional”.

### Screening for UCC

In our sample, 90 ± 3% of French women aged 25–40 years in the Nord-Pas-de-Calais region had been screened for UCC by a Pap smear in the previous 3 years. In bivariate analysis, women at risk of not being screened were the youngest, HPV vaccinated, non-heterosexual, living alone, having gynecologic follow-up by their general practitioner, and not having regular gynecologic follow-up. Sensitivity analysis by age subgroup showed that the only risk factor that remained significantly correlated with non-screening regardless of age group was lack of regular gynecologic follow-up.

Regarding the association between vaccination and screening, the Australian studies showed both a negative [22] and a positive [19] effect of vaccination on screening. The data linkage process was different in the two studies, and the positive effect was mainly found in the most recent study (2019 vs 2014). Overall, a positive association between HPV vaccination and HPV screening has been reported in the literature from Sweden in 2015 [20], the United States [18, 36], Italy [21], Japan [37], and the United Kingdom and Denmark [14, 15]. In a Canadian study, participation in screening was significantly lower among vaccinated women than among unvaccinated women, but as in our study, this difference was no longer significant after adjustment for age [38]. For the time being, it seems difficult to obtain an answer in France, as HPV vaccination only started in 2007 and its indications are still evolving, first for women and then for the whole population. For example, to study a cohort of 35-year-old women (covered by screening for 10 years) potentially vaccinated in 2013–2015, we will have to wait until 2037.

Regarding participation in screening, in our study the overall screening coverage and the coverage by age group were higher than those reported at departmental and national level for the period 2017–2020 [39]. This could be explained by a bias in patient volunteering and an overrepresentation of women in couples. In addition, other studies that used a declarative method to assess participation in screening found high participation rates [16, 40] of around 90%.

Several sociodemographic factors are associated with screening in the literature. Women who live with a partner, have a higher income, have supplementary private health insurance, and have consulted a gynecologist in the past 12 months were most likely to undergo screening [41]. A significant association has been found between the socioeconomic level of the neighborhood surrounding the primary care practice and participation in UCC screening at that practice [42]. There was also a significant relationship between the density of gynecologists within 5 km and between 20 and 40 km of the GP practice, with a greater effect for less than five kilometers [43]. In our study, we only knew the socio-professional category, age, and department. Demographic and social data such as income, supplementary health insurance, distance to a general practitioner's office and distance to a gynecologist's office were not collected. However, we found the effect of gynecological follow-up on a declarative basis. Women in France are encouraged to have an annual gynecological follow-up consultation 1) to discuss contraception and sexually transmitted infections (STI), 2) to propose recommended screening. Self-sampling of STI is annually proposed for women under 25. Annual clinical breast monitoring is proposed from age 25. Cervical cancer screening is proposed every 3 years from age 25 and then every 5 years from age 30 to 65.

Knowledge of the women has been identified as a risk factor for poor screening in Ethiopian police officers [44] but was not a risk factor in our population, probably because women in our sample were predominantly screened.

### Strengths and limitations

One of the strengths of this study was its originality and the fact that it was conducted in a primary care setting. To our knowledge, it was the first of its kind in France. The doctors were randomly selected, which allowed us to randomly distribute the questionnaire throughout the Nord and Pas-de-Calais regions. The fact that we distributed our questionnaire electronically was also a strength. Indeed, several studies have shown that the validity of methods based on an electronic format is equivalent to that of paper questionnaires [45, 46]. The posters and QR code cards allowed women to respond while waiting in the waiting room and later.

There was a selection bias because the doctors recruited were likely to be more sensitive to and interested in the topic of the study. There was also a voluntary selection bias because only motivated patients responded. This double selection bias could lead to an over-representation of health-conscious women amplified by an over-representation of voluntary doctors having a greater interest in cervical cancer. It could present an overly optimistic view of preventive measures. In addition, women who did not have a telephone or Internet connection and those with little or no access to health care were unable to respond, although these may be the most disadvantaged populations and those furthest from the health care system, with low vaccination and screening rates. There was a recall bias for both vaccination and screening due to the self-reporting mode, as reported in the literature [47]. There was a social desirability bias, particularly as the questionnaire was displayed in a doctor's surgery where anonymity was limited. For future research, we should consider stratified sampling, and using administrative date instead of self-reports.

## Conclusion

In young women, participation in HPV vaccination and UCC screening is enhanced by medical information from the family doctor and regular gynecological check-ups. The internal validity of this study was strengthened by its multicenter design with random selection of practices, but limited by selection bias (recruitment was done in the practices by patients interested in the topic), recall bias (the questionnaire was retrospective), and survey bias (the questionnaire was anonymous). The main drawback is the relatively recent promotion of HPV vaccination in France. It would be advisable to repeat this study starting in 2037 to obtain robust results on the effect of vaccination on screening.

## Data Availability

The datasets used and/or analyzed during the current study are available from the corresponding author on reasonable request.

## Abbreviations

GP: General practitioner
HAS: High authority in health (French)
HPV: Human papilloma viruses
INCa: (French) National institute of cancer
UCC: Uterine cervical cancer
WHO: World health organization

## References

[1] Sung H, Ferlay J, Siegel RL, Laversanne M, Soerjomataram I, Jemal A (2021). Global Cancer Statistics 2020: GLOBOCAN Estimates of Incidence and Mortality Worldwide for 36 Cancers in 185 Countries. CA Cancer J Clin.

[2] Hamers FF, Woronoff A-S (2019). Cancer du col de l’utérus en France : tendances de l’incidence et de la mortalité jusqu’en 2018. Bull Epidémiol Hebd.

[3] Bajos N, Bozon M, Beltzer N, Laborde C, Andro A, Ferrand M (2010). Changes in sexual behaviours: from secular trends to public health policies. AIDS Lond Engl.

[4] Beck F, Richard J-B. Les comportements de santé des jeunes: analyses du Baromètre santé 2010. Saint-Denis: INPES éd; 2013. https://www.santepubliquefrance.fr/docs/les-comportements-de-sante-des-jeunes-analyses-du-barometre-sante-2010.

[5] Cancer du col de l’utérus. https://www.who.int/fr/news-room/fact-sheets/detail/cervical-cancer. Accessed 17 May 2022.

[6] Urrutia M-T, Araya A-X, Gajardo M, Chepo M, Torres R, Schilling A (2023). Acceptability of HPV Vaccines: A Qualitative Systematic Review and Meta-Summary. Vaccines.

[7] Gorgone M, Squeri A, Cuffari S, Fauci VL, Giunta I, Calderone S (2023). Rates of Primary and Secondary Prevention of Cervical Cancer: A Study in a Province in the South of Italy. Vaccines.

[8] Jin SW, Lee Y, Lee S, Jin H, Brandt HM (2023). Factors Associated with College Students’ Human Papillomavirus (HPV) Vaccination and Preferred Strategies for Catch-Up Vaccine Promotion: A Mixed-Methods Study. Vaccines.

[9] Lubeya MK, Chibwesha CJ, Mwanahamuntu M, Mukosha M, Vwalika B, Kawonga M (2024). Determinants of the Implementation of Human Papillomavirus Vaccination in Zambia: Application of the Consolidated Framework for Implementation Research. Vaccines.

[10] Li J, Kang L-N, Li B, Pang Y, Huang R, Qiao Y-L (2015). Effect of a group educational intervention on rural Chinese women’s knowledge and attitudes about human papillomavirus (HPV) and HPV vaccines. BMC Cancer.

[11] Lefevere E, Hens N, Theeten H, Van den Bosch K, Beutels P, De Smet F (2011). Like mother, like daughter? Mother’s history of cervical cancer screening and daughter’s Human Papillomavirus vaccine uptake in Flanders (Belgium). Vaccine.

[12] Lutringer-Magnin D, Cropet C, Barone G, Canat G, Kalecinski J, Leocmach Y (2013). HPV vaccination among French girls and women aged 14–23 years and the relationship with their mothers’ uptake of Pap smear screening: a study in general practice. Vaccine.

[13] Tron A, Schlegel V, Gilberg S, Partouche H (2021). Obstacles et facilitateurs du vaccin contre le papillomavirus : une étude qualitative auprès de 26 médecins généralistes français. Infect Dis Now.

[14] Hestbech MS, Gyrd-Hansen D, Kragstrup J, Siersma V, Brodersen J (2016). How does HPV vaccination status relate to risk perceptions and intention to participate in cervical screening? a survey study. BMC Public Health.

[15] Beer H, Hibbitts S, Brophy S, Rahman MA, Waller J, Paranjothy S (2014). Does the HPV vaccination programme have implications for cervical screening programmes in the UK?. Vaccine.

[16] Bernard E, Saint-Lary O, Haboubi L, Le Breton J (2013). Dépistage du cancer du col de l’utérus : connaissances et participation des femmes. Santé Publique.

[17] Taniguchi M, Ueda Y, Yagi A, Ikeda S, Endo M, Tomimatsu T (2019). Cervical cancer screening rate differs by HPV vaccination status: An interim analysis. Vaccine.

[18] Ba DM, McCall-Hosenfeld JS, Ssentongo P, Chinchilli VM, Agbese E, Liu G (2021). Cervical cancer screening varies by HPV vaccination status among a National Cohort of privately insured young women in the United States 2006–2016. Medicine (Baltimore).

[19] Australian Institute of Health and Welfare. Analysis of cervical cancer and abnormality outcomes in an era of cervical screening and HPV vaccination in Australia. Cancer Ser. 2019;126:1–144.

[20] Herweijer E, Feldman AL, Ploner A, Arnheim-Dahlström L, Uhnoo I, Netterlid E (2015). The Participation of HPV-Vaccinated Women in a National Cervical Screening Program: Population-Based Cohort Study. PLoS ONE.

[21] Del Mistro A, Battagello J, Weis L, Bressan V, Selle V, Ramigni M (2021). A Retrospective Cohort Study of Young Women Spontaneously Choosing to Be Vaccinated against HPV: Outcomes from Their First Cervical Cancer Screening Test. Viruses.

[22] Budd AC, Brotherton JML, Gertig DM, Chau T, Drennan KT, Saville M (2014). Cervical screening rates for women vaccinated against human papillomavirus. Med J Aust.

[23] Isserlis L (1918). On the Value of a Mean as Calculated from a Sample. J R Stat Soc.

[24] The jamovi project (version 2.4) [computer software]. https://www.jamovi.org/.

[25] R Core Team (2022). R: A Language and environment for statistical computing. (Version 4.1) [Computer software]. https://cran.r-project.org.

[26] Medistica. pvalue.io, a Graphic User Interface to the R statistical analysis software for scientific medical publications. 2021. Available on: https://www.pvalue.io/fr.

[27] Le vaccin contre les HPV il est vraiment sûr docteur ? Communiqué de Presse. Institut National du Cancer. 2022. https://www.e-cancer.fr/Presse/Dossiers-et-communiques-de-presse/LE-VACCIN-CONTRE-LES-HPV-IL-EST-VRAIMENT-SUR-DOCTEUR-L-Institut-national-du-cancer-aux-cotes-des-professionnels-de-sante-dans-l-information-de-leur-patientele.

[28] Crosby RA, Casey BR, Vanderpool R, Collins T, Moore GR (2011). Uptake of Free HPV Vaccination Among Young Women: A Comparison of Rural Versus Urban Rates. J Rural Health Off J Am Rural Health Assoc Natl Rural Health Care Assoc.

[29] Staras SAS, Vadaparampil ST, Haderxhanaj LT, Shenkman EA (2010). Disparities in Human Papillomavirus Vaccine Series Initiation Among Adolescent Girls Enrolled in Florida Medicaid Programs, 2006–2008. J Adolesc Health Off Publ Soc Adolesc Med.

[30] Reiter PL, Cates JR, McRee A-L, Gottlieb SL, Shafer A, Smith JS (2010). Statewide HPV Vaccine Initiation Among Adolescent Females in North Carolina. Sex Transm Dis.

[31] Lasset C, Kalecinski J, Régnier V, Barone G, Leocmach Y, Vanhems P (2014). Practices and opinions regarding HPV vaccination among French general practitioners: evaluation through two cross-sectional studies in 2007 and 2010. Int J Public Health.

[32] Lions C, Pulcini C, Verger P (2013). Papillomavirus vaccine coverage and its determinants in South-Eastern France. Médecine Mal Infect.

[33] Rondy M, van Lier A, van de Kassteele J, Rust L, de Melker H (2010). Determinants for HPV vaccine uptake in the Netherlands: A multilevel study. Vaccine.

[34] Huon J-F, Grégoire A, Meireles A, Lefebvre M, Péré M, Coutherut J (2020). Evaluation of the acceptability in France of the vaccine against papillomavirus (HPV) among middle and high school students and their parents. PLoS ONE.

[35] Collange F, Fressard L, Pulcini C, Sebbah R, Peretti-Watel P, Verger P (2016). General practitioners’ attitudes and behaviors toward HPV vaccination: A French national survey. Vaccine.

[36] Price RA, Koshiol J, Kobrin S, Tiro JA (2011). Knowledge and Intention to Participate in Cervical Cancer Screening after the Human Papillomavirus Vaccine. Vaccine.

[37] Taniguchi M, Ueda Y, Yagi A, Miyoshi A, Tanaka Y, Minekawa R (2021). Disparity of Cervical Cancer Risk in Young Japanese Women: Bipolarized Status of HPV Vaccination and Cancer Screening. Vaccines.

[38] Sauvageau C, Gilca V, Ouakki M, Kiely M, Coutlée F, Mathieu-Chartier S (2021). Sexual behavior, clinical outcomes and attendance of cervical cancer screening by HPV vaccinated and unvaccinated sexually active women. Hum Vaccines Immunother.

[39] Dépistage du cancer du col de l’utérus : données 2017–2019. https://www.santepubliquefrance.fr/les-actualites/2021/depistage-du-cancer-du-col-de-l-uterus-donnees-2017-2019#:~:text=58%2C2%25%20des%20femmes%20de,du%20col%20de%20l'ut%C3%A9rus. Accessed 26 May 2021.

[40] Oussaid N, Lutringer-Magnin D, Barone G, Haesebaert J, Lasset C (2013). Factors associated with Pap smear screening among French women visiting a general practitioner in the Rhône-Alpes region. Rev Epidemiol Sante Publique.

[41] Barré S, Massetti M, Leleu H, Catajar N, de Beis F (2017). Caractérisation des femmes ne réalisant pas de dépistage du cancer du col de l’utérus par frottis cervico-utérin en France. Bull Epidémiol Hebd.

[42] Serman F, Favre J, Deken V, Guittet L, Collins C, Rochoy M (2020). The association between cervical cancer screening participation and the deprivation index of the location of the family doctor’s office. PLoS ONE.

[43] Quersin F, Serman F, Favre J, Rochoy M, Descamps A, Gers E (2022). Participation rate in cervical cancer screening in general practice related to the proximity of gynecology care facilities: A 3 year follow-up cohort study. Front Public Health.

[44] Misgun T, Demissie DB (2023). Knowledge, practice of cervical cancer screening and associated factors among women police members of Addis Ababa police commission Ethiopia. BMC Cancer.

[45] Campbell N, Ali F, Finlay AY, Salek SS (2015). Equivalence of electronic and paper-based patient-reported outcome measures. Qual Life Res.

[46] Muehlhausen W, Doll H, Quadri N, Fordham B, O’Donohoe P, Dogar N (2015). Equivalence of electronic and paper administration of patient-reported outcome measures: a systematic review and meta-analysis of studies conducted between 2007 and 2013. Health Qual Life Outcomes.

[47] Yamaguchi M, Sekine M, Kudo R, Adachi S, Ueda Y, Miyagi E (2018). Differential misclassification between self-reported status and official HPV vaccination records in Japan: Implications for evaluating vaccine safety and effectiveness. Papillomavirus Res.

